# AOZORA study: 3-year interim analysis of safety and joint health in pediatric people with hemophilia A receiving emicizumab prophylaxis

**DOI:** 10.1016/j.rpth.2025.103228

**Published:** 2025-10-21

**Authors:** Midori Shima, Hideyuki Takedani, Kaoru Kitsukawa, Masashi Taki, Akira Ishiguro, Chiai Nagae, Azusa Nagao, Daisuke Nosaka, Yui Kyogoku, Hiroki Oki, Keisuke Iwasaki, Keiji Nogami

**Affiliations:** 1 Thrombosis and Hemostasis Research Center, Nara Medical University, Nara, Japan; 2 Department of Rehabilitation, National Hospital Organization Tsuruga Medical Center, Fukui, Japan; 3 Department of Radiology, Chiba University School of Medicine, Chiba, Japan; 4 Department of Pediatrics, St Marianna University School of Medicine, Kanagawa, Japan; 5 Division of Hematology, National Center for Child Health and Development, Tokyo, Japan; 6 Department of Blood Coagulation, Ogikubo Hospital, Tokyo, Japan; 7 Medical Affairs Division, Chugai Pharmaceutical Co, Ltd, Tokyo, Japan; 8 Clinical Development Department, Chugai Pharmaceutical Co, Ltd, Tokyo, Japan; 9 Biometrics Department, Chugai Pharmaceutical Co, Ltd, Tokyo, Japan; 10 Department of Paediatrics, Nara Medical University, Nara, Japan

**Keywords:** antibodies, Emicizumab, hemarthrosis, hemophilia A, pediatrics

## Abstract

**Background:**

Recurrent joint bleeding in people with hemophilia A (PwHA) can cause hemophilic arthropathy, resulting in limited movement and chronic pain. Emicizumab is a bispecific monoclonal antibody bridging activated factor (F)IX and FX to substitute for deficient activated FVIII in PwHA, thereby improving hemostasis.

**Objectives:**

This 3-year interim analysis of the ongoing, open-label, phase IV AOZORA study (jRCT1080224629) analyzes medium-term safety and joint health in pediatric PwHA without FVIII inhibitors receiving emicizumab.

**Methods:**

PwHA aged <12 years with severe hemophilia A without FVIII inhibitors were eligible. Participants entered AOZORA as emicizumab-naïve or having previously initiated emicizumab during the HOHOEMI study. Endpoints included safety, and joint health, as assessed by magnetic resonance imaging and Hemophilia Joint Health Score (HJHS). Participants will receive emicizumab for 6 years.

**Results:**

A total of 30 male PwHA were enrolled. Data cutoff was the last day of week 145 for each participant. Median (range) age was 4.2 (0.7-11.1) years, and 27 of the 30 (90.0%) had received prior FVIII prophylaxis. The emicizumab safety profile was confirmed. No thrombotic events/microangiopathies occurred. All joints with synovial hypertrophy and hemosiderin resolved or improved by week 145. HJHS remained at 0 from week 1 to week 145 for 18 (66.7%) participants; overall, there was no worsening trend in HJHS over time. Model-based annualized bleeding rate (95% CI) for treated bleeds was 3.6 (2.04-6.46) prior to emicizumab and 0.8 (0.47-1.22) after receiving emicizumab.

**Conclusion:**

Emicizumab is well tolerated and appears to maintain or improve joint health in pediatric PwHA.

## Essentials

• Long-term joint health data in people with hemophilia A receiving emicizumab are lacking.
• AOZORA is evaluating long-term safety and joint health with emicizumab in pediatric hemophilia A.
• A 3-year interim analysis confirmed the safety profile of emicizumab.
• Magnetic resonance imaging data suggest emicizumab can maintain joint health and reduce pathologic changes over time.

## 1 Introduction

Hemophilia A (HA) is a congenital bleeding disorder characterized by a deficiency or dysfunction of factor (F)VIII, leading to recurrent bleeding, mainly into the joints [[1], [2], [3]]. Joint bleeds in young children typically start to occur once they begin to crawl and walk and can be a key indicator of the clinical severity of the disease [3,4]. The majority of treated bleeds occur in the joints, primarily the ankle, knee, and elbow [3]. Recurrent joint bleeding leads to hemosiderin deposition, synovial degeneration, and inflammation, which, if they progress further, result in irreversible osteochondral deformities and destruction [5,6].

The risk of joint damage in people with hemophilia A (PwHA) can be reduced by the use of effective prophylaxis early in life [1,7]; however, it has been demonstrated that joint bleeds still occur, even with regular FVIII replacement [[7], [8], [9], [10]]. Despite the control of bleeding, some patients have worsening joint conditions, suggesting the possibility of subclinical bleeding [11,12].

Emicizumab is a bispecific monoclonal antibody that bridges activated FIX and FX to substitute for deficient activated FVIII [13] and is used as prophylactic treatment to prevent or reduce the frequency of bleeding episodes in both adults and children with HA [14,15]. Emicizumab has been demonstrated to be an effective and well-tolerated option, with safety data available for 6 years of treatment [2,[16], [17], [18], [19], [20], [21], [22], [23], [24], [25]]. Emicizumab has been shown to maintain stable trough levels over time, offering continued protection against bleeding in PwHA [[20], [21], [22], [23]]; however, there is a lack of long-term data on joint health in children receiving emicizumab prophylaxis through to adulthood.

AOZORA (jRCT1080224629, https://jrct.niph.go.jp) is a postmarketing clinical study designed to evaluate the long-term safety and joint-health effects of emicizumab in people with severe hemophilia A (PwSHA) aged <12 years, without FVIII inhibitors [26]. In AOZORA, joint assessment using magnetic resonance imaging (MRI) was conducted to detect early joint changes. In this study, the data from a planned interim analysis of AOZORA are reported, constituting almost 3 years of follow-up.

## 2 Methods

### 2.1 Study design and participants

**Figure 1:**
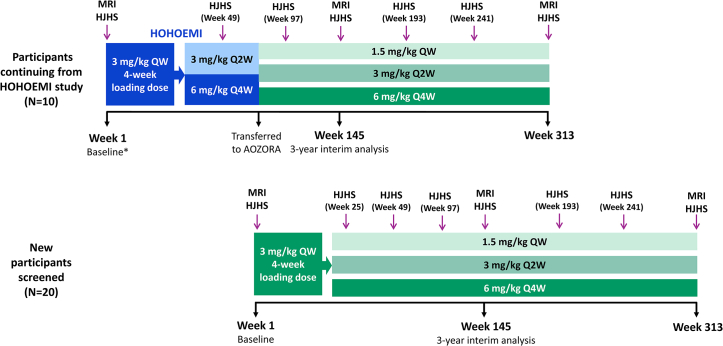
Summary of AOZORA study design. ∗At the start of the HOHOEMI study. During AOZORA, it was permitted for the dosing regimen to be changed between any of the three approved regimens, at the discretion of the treating physician. HJHS, Hemophilia Joint Health Score; MRI, magnetic resonance imaging; QW, weekly; Q2W, every 2 weeks; Q4W, every 4 weeks.

AOZORA is an ongoing, multicenter, open-label, phase IV clinical trial, including 10 Japanese institutions to evaluate the efficacy and safety of emicizumab in Japanese pediatric PwSHA without FVIII inhibitors. The protocol has been previously published [26]. The study has a planned long-term follow-up period of 6 years (Figure 1). AOZORA aimed to recruit approximately 30 pediatric PwSHA who are either emicizumab-naïve or previously initiated emicizumab treatment as part of the phase III HOHOEMI study [2].

Eligible participants are <12 years, with body weight of >3 kg, and a diagnosis of severe congenital HA (endogenous FVIII level <1%), together with negative results for FVIII inhibitors within 8 weeks prior to study enrollment. Key exclusion criteria included the presence of bleeding disorders other than congenital HA; currently undergoing immune tolerance induction (ITI) therapy; previous or current treatment for, or signs of, thromboembolic disease; and prior receipt of emicizumab (with the exception of participants transferring from HOHOEMI).

### 2.2 Interventions and study procedures

Emicizumab was administered subcutaneously as 1 of 3 approved maintenance regimens: 1.5 mg/kg weekly, 3.0 mg/kg every 2 weeks, or 6.0 mg/kg every 4 weeks (Figure 1). The observation period for each participant was defined as the date of the first emicizumab dose (in HOHOEMI or at the start of AOZORA) until the date of the last observation, the date of the final safety assessment (performed 24 weeks after the last dose for any participants who discontinued treatment), the date of withdrawal of informed consent, or the date of loss to follow-up.

Short-term FVIII prophylaxis was permitted in case of surgical intervention. Additionally, FVIII products could be used episodically to treat breakthrough bleeds at the discretion of the treating physician. Concomitant treatments that were prohibited include other investigational drugs, long-term FVIII prophylaxis, and situational prophylaxis (eg, for bleed prevention prior to sports or rehabilitation activities).

### 2.3 Study endpoints

#### 2.3.1 Primary endpoints

The long-term safety of emicizumab in pediatric PwSHA was assessed by recording adverse events (AEs), AEs leading to discontinuation of emicizumab, AEs of special interest (ie, hypersensitivity reactions, anaphylaxis and anaphylactoid reactions, thromboembolic events, and thrombotic microangiopathies), physical examination findings, laboratory test abnormalities and development of FVIII inhibitors.

For the evaluation of joints in this study, changes in joints from baseline (week 1) to week 313 were assessed using MRI and Hemophilia Joint Health Score (HJHS). MRI imaging protocols were predefined for the study (Supplementary Table 1), and all participating institutions followed the standardized protocols for MRI acquisition. MRI scans of bilateral knee and ankle joints were performed in a single examination and by acquiring T2∗-weighted images in both sagittal and coronal planes. The detailed imaging parameters were as follows: for 1.5 Tesla (T) MRI systems, repetition time (TR) was 400 to 600 ms, echo time (TE) was 13.8 ms (recommended), and flip angle was 20° to 30°. For 3.0 T MRI systems, TR was 400 to 600 ms, TE was 6.9 or 9.2 ms (recommended), and flip angle was 20° to 25°. Slice thickness was set at 5 mm or less. The field-of-view was optimized according to the subject’s body size, with reference values of approximately 150 mm for infants and toddlers, and up to approximately 180 mm for school-aged children and older, depending on their physical build. Since this study targeted pediatric patients, evaluation of ankle and knee joints, which are subject to weight-bearing stress, was prioritized, and MRI imaging of elbow joints was not performed from the perspective of reducing burden on participants. Joint health was evaluated using the International Prophylaxis Study Group (IPSG) MRI scale, which allocates 9 points for soft tissue changes (effusion/hemarthrosis, synovial hypertrophy, and hemosiderin deposition) and 8 points for osteochondral changes (surface erosions, subchondral cysts, and cartilage degradation) [27,28]. MRI images were evaluated through a concurrent review process where 2 reviewers observed and assessed the images cooperatively. A score of 0 represents no joint changes, while a score of ≥1 represents pathologic joint changes. As it is difficult to distinguish hemosiderin deposition from synovial hypertrophy, they were scored together [12]. MRI assessments were planned for baseline (week 1), week 145, and week 313.

The HJHS v2.1 was used for the assessment of joint function [29,30]. Individual scores for the elbow, ankle, and knee joints (index joints) were determined through physical examination. The sum of these joint scores, along with a global gait score, was then combined to calculate the overall total HJHS. HJHS was conducted at each facility, and evaluations were performed only by personnel who had received training in assessment. For participants who started emicizumab treatment for the first time in AOZORA, HJHS assessments were conducted at baseline, at week 25, week 49, and every 48 weeks thereafter. For participants continuing from the HOHOEMI study, HJHS assessments were conducted at baseline and every 48 weeks thereafter [26].

#### 2.3.2 Other endpoints including exploratory

All treated bleeds throughout the study were captured. Exploratory endpoints included activities and activity-related bleeds during emicizumab prophylaxis. Bleeding events were subjectively collected as patient-reported outcomes, with caregivers documenting these events in paper-based questionnaires that were submitted to the facilities. A bleeding event was considered as a treated bleed if coagulation factors were administered subsequent to the manifestation of signs and symptoms of bleeding (eg, pain and swelling). Data on bleeds that occurred in the 24 weeks prior to study entry were collected retrospectively.

### 2.4 Statistical analyses

The required sample size was not determined based on statistical hypothesis testing. A sample size of approximately 30 PwSHA was selected based on study feasibility and the number of participants required to assess the effects of long-term emicizumab on joints.

Data were collected via electronic data capture by means of electronic case report forms. Participant- and caregiver-reported outcome data (bleeds, breakthrough bleed treatment, and activities) were collected using paper questionnaires, and data were entered into the electronic data capture system by study site staff. Annualized bleed rates (ABRs) for each participant were calculated by counting the number of bleeds reported for a participant and dividing by the efficacy analysis period. Model-based ABRs were estimated using negative binomial regression. Mean and median ABRs for the overall study population were also calculated.

### 2.5 Ethical considerations

This study was approved by a local ethical review committee and conducted in compliance with the Declaration of Helsinki, the study protocol, the standards stipulated in Paragraph 3 of Article 14 and Article 80-2 of the Pharmaceuticals, Medical Devices and Other Therapeutic Products Act, the Ministerial Ordinance on Good Clinical Practice, and the Ministerial Ordinance on Good Post-marketing Study Practice.

## 3 Results

### 3.1 Study population

A total of 30 participants were enrolled into the study, 10 of whom had previously initiated emicizumab as part of the phase III HOHOEMI study and 20 who were newly enrolled into the AOZORA study (Table 1). The present analysis includes data collected at baseline (week 1) through to the last day of week 145 for each participant. Three participants discontinued from the study prior to week 145; reasons included refusal of MRI (*n* = 1), moving (*n* = 1), and increased caregiver burden (*n* = 1). At the time of data cutoff, 27 participants continued in the AOZORA study.

**Table 1:** Participant demographics and baseline characteristics.

Characteristic	Participants entering from HOHOEMI (*n* = 10)	Participants newly entering AOZORA (*n* = 20)	Total participants (*N* = 30)
Age (y)	5.9 (1.5-10.7)	3.8 (0.7-11.1)	4.2 (0.7-11.1)
Age category (y)			
0 to <2 y	2 (20.0)	4 (20.0)	6 (20.0)
2 to <6 y	3 (30.0)	9 (45.0)	12 (40.0)
6 to <12 y	5 (50.0)	7 (35.0)	12 (40.0)
Male	10 (100)	20 (100)	30 (100)
Weight (kg)	19.4 (9.5-35.6)	15.9 (7.3-63.1)	16.3 (7.3-63.1)
Treatment regimen with coagulation factor products prior to enrollment			
Episodic FVIII	0 (0)	2 (10.0)	2 (6.7)
Prophylactic FVIII	10 (100)	17 (85.0)	27 (90.3)
Previously untreated	0	1 (5.0)	1 (3.3)
Participants previously treated with ITI therapy	2 (20.0)	3 (15.0)	5 (16.7)
Duration of ITI (y)	0.8 (0.5-1.1)	2.3 (0.6-5.3)	1.2 (0.5-5.3)
Period from end of ITI to emicizumab initiation (y)	3.9 (0.4-7.4)	3.2 (1.2-5.0)	3.2 (0.4-7.4)
Participants with target joints	1 (10.0)^a^	0 (0)	1 (3.3)^a^

Values are n (%) or median (range).

FVIII, factor VIII; ITI, immune tolerance induction.

a Target joint was 1 left knee in 1 individual. Target joints were defined as a major joint (eg, hip, elbow, wrist, shoulder, knee, or ankle) in which at least 3 bleeds had occurred within the last 24 weeks prior to emicizumab initiation.

The median (range) age of the 30 enrolled participants was 4.2 (0.7-11.0) years, and all were males. The majority (*n* = 27; 90.0%) of participants had previously been treated with FVIII prophylaxis, while 2 (6.7%) had been treated with episodic FVIII and 1 (3.3%) was previously untreated. Of the 27 participants who had previously been treated with FVIII prophylaxis, 15 were on standard half-life (SHL) products and 10 were on extended half-life products; data were not available for the remaining 2 participants. The majority of participants had initiated FVIII prophylaxis before 2 years of age and had continued treatment for more than 1 year. Of the 30 participants, 5 were previously treated with ITI therapy (2 from HOHOEMI and 3 newly enrolled participants). Median (range) duration of ITI was 1.2 (0.5-5.3) years. One of the 30 participants had a target joint (left knee) at baseline; the participant had received prophylaxis with SHL FVIII prior to emicizumab administration and had no history of ITI.

### 3.2 Safety

Of 30 participants, 29 (96.7%) study participants experienced at least 1 AE, with a total of 404 events reported (Table 2 and Supplementary Table 2). Five participants experienced a total of 7 treatment-related AEs: 6 injection-site reactions and 1 case of anemia, where hemoglobin levels decreased from 14.1 to 13.1 g/dL, with no clinical symptoms.

**Table 2:** Safety summary during 145 wk of prophylaxis.

Adverse events	Total participants (*N* = 30)
Total No. of AEs	404
Participants with ≥1 AE	
Any AE	29 (96.7)
AE leading to withdrawal from treatment	0
AE leading to dose modification/interruption	0
Fatal AE	0
Grade 3-5 AE	5 (16.7)^a^
Emicizumab-related AE	5 (16.7)
Injection-site reaction	5 (16.7)
Anemia	1 (3.3)
Any SAE	5 (16.7)
Emicizumab-related SAE	0

Values are n (%).

AE, adverse event; SAE, serious adverse event.

a Five participants experienced 7 events: grade 3 AEs including posttraumatic pain (*n* = 1); soft tissue hemorrhage (*n* = 1); hemophilic arthropathy (*n* = 1) and hemarthrosis (*n* = 2); grade 4 AEs including skull fracture (*n* = 1) and subdural hematoma (*n* = 1).

FVIII inhibitors developed in 1 participant who had received SHL FVIII prophylaxis before starting emicizumab. When a joint bleed occurred on day 898, FVIII concentrates were administered 22 times over 13 days; FVIII inhibitors were later detected for the first time (28.9 BU/mL). After the development of FVIII inhibitors, this participant continued to receive emicizumab and remained in the study. Additional details have been previously reported [31].

Seven serious AEs were reported in 5 participants; none were considered emicizumab-related treatment: post-traumatic pain (*n* = 1); soft tissue hemorrhage (*n* = 1); hemophilic arthropathy (*n* = 1); 2 cases of hemarthrosis (*n* = 1); and a skull fracture and subdural hematoma that occurred concurrently (*n* = 1). All serious AEs were recovered/resolved at the time of the data cutoff, following administration of FVIII products. There were no fatal AEs and no AE resulted in withdrawal from treatment or dose modification/interruption. No thrombotic events or thrombotic microangiopathies were reported.

### 3.3 MRI findings

**Figure 2:**
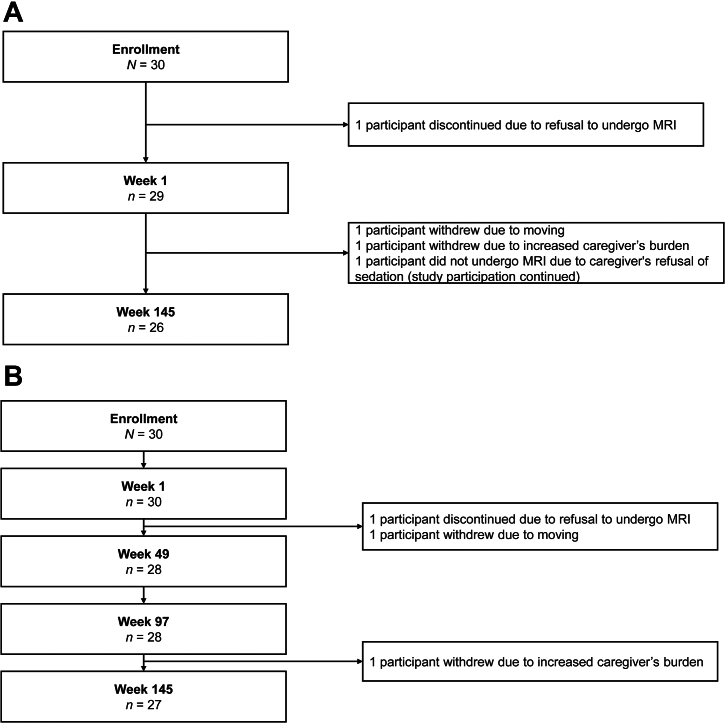
(A) Flow chart of participants who underwent MRI. (B) Flow chart of participants who underwent HJHS. HJHS, Hemophilia Joint Health Score; MRI, magnetic resonance imaging.

**Figure 3:**
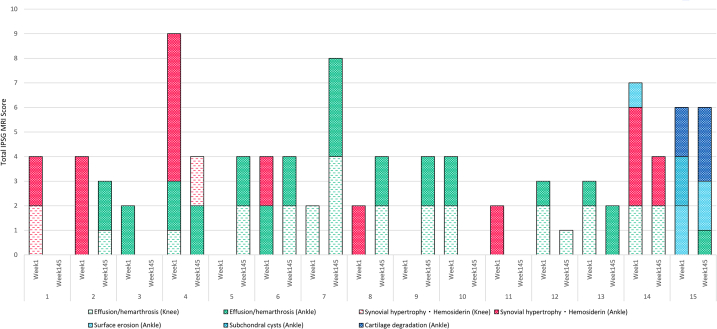
Changes in the interindividual IPSG MRI score (n = 15). Participant 4 developed synovial hypertrophy and hemosiderin deposition in 1 knee joint by week 145. Participant 10 and participants 5-9 had the same effusion/hemarthrosis scores in all 4 joints at week 1 and week 145, respectively. Participants 14 and 15 also had osteochondral changes. IPSG, International Prophylaxis Study Group; MRI, magnetic resonance imaging.

MRI data were obtained from 29 participants at week 1 (data could not be obtained for 1 participant who discontinued the study due to refusal of MRI) and 26 participants at week 145. MRI data were missing for 3 additional participants at week 145 due to withdrawal from the study (*n* = 2) and refusal of sedation for MRI by the caregiver (*n* = 1) (Figure 2A). Of 26 participants, 11 (42.3%) had an IPSG MRI score of zero at both week 1 and week 145. Changes in IPSG MRI score for 15 participants from week 1 to week 145 are shown in Figure 3. Among these 15 participants, 9 participants showed score improvements, 2 participants maintained the same score, and 4 participants experienced an increase in score. Notably, all 7 participants who presented with synovial hypertrophy and hemosiderin deposition at week 1 demonstrated either complete resolution or improvement of these findings by week 145. In participant 4, while the synovial hypertrophy and hemosiderin deposition observed in the ankle joint at week 1 had resolved by week 145, new findings of synovial hypertrophy and hemosiderin deposition were observed in the knee joint at week 145. No treated bleeds were reported in this knee joint between week 1 and week 145. Additionally, all 4 participants (participants 5, 7, 8, and 9) showing increased scores from week 1 to week 145 demonstrated an increase in effusion/hemarthrosis. No treated joint bleeds were reported in these participants from week 1 to week 145. Furthermore, among the 12 participants with 28 joints showing soft tissue findings at week 1, only 2 participants experienced a traumatic bleeding episode, in 1 joint each, by week 145; the IPSG MRI scale scores for soft tissue in these joints had improved by week 145.

Osteochondral changes were observed in 2 participants (participant 14 and 15). Participant 14, who was 8 years old at week 1, had a score of 1 (bone erosion: 1) in the ankle joint; this observation was not noted at week 145. Although this participant experienced 1 traumatic bleeding episode in the same joint between week 1 and week 145, the score for synovial hypertrophy and hemosiderin improved. Participant 15, who was 9 years old at week 1, had a history of ITI and presented with hemophilic arthropathy in the left ankle joint at the time of study enrollment. The IPSG MRI score in participant 15 was 6 (bone erosion, 2; subchondral cyst, 2; cartilage degradation, 2) in the left ankle joint at week 1, which changed to 6 (effusion/hemarthrosis, 1; bone erosion, 2; cartilage degradation, 3) in the same joint at week 145. This participant experienced a total of 2 bleeding episodes (1 traumatic and 1 spontaneous) by week 145.

Among the 5 participants with a history of ITI, MRI findings at week 1 were observed in only 2 participants: one being the previously described case, and the other exhibiting effusion in the ankle joint. Participants with prior ITI had initiated therapy at 1 to 2 years of age, indicating early intervention with FVIII prophylaxis. The condition of the joints appeared similar between participants with and without ITI history.

### 3.4 Hemophilia Joint Health Score

**Figure 4:**
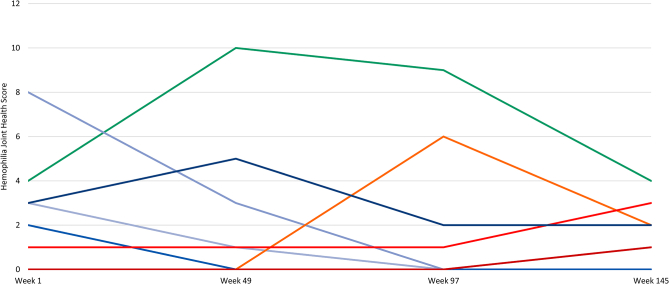
Changes in HJHS for each participant. Each line represents the total HJHS for an individual participant. Of the 9 participants with score changes, 1 was due to consent withdrawal, with only week 1 results available. HJHS, Hemophilia Joint Health Score.

All 30 participants had HJHS data available at week 1, with 27 (90.0%) having data at week 145 (Figure 2B). Changes in mean HJHS over time for total score are shown in Table 3. For total score, there was an overall decrease (improvement) from week 1 to week 145. The HJHS score remained at 0 for 18 (66.7%) participants from week 1 to week 145. In the other 11 participants, 5 had a decrease in HJHS score, 3 had an increase in HJHS score, 1 participant had no change in HJHS scores from week 1 to week 145, and 2 individuals withdrew consent, so data later than week 1 were unavailable (Figure 4).

**Table 3:** Change in mean HJHS scores over time.

Total (all joints + global gait)	Week 1 (*N* = 30)	Week 49 (*n* = 28)	Week 97 (*n* = 28)	Week 145 (*n* = 27)
Mean (SD)	0.90 (1.79)	0.71 (2.12)	0.64 (2.02)	0.44 (1.05)
Median (range)	0.0 (0.0-8.0)	0.0 (0.0-10.0)	0.0 (0.0-9.0)	0.0 (0.0-4.0)

Lower HJHS indicates better joint health.

HJHS, Hemophilia Joint Health Score v2.1.

### 3.5 Bleeding rate

The model-based ABR for treated bleeds decreased from 3.6 (95% CI, 2.04-6.46) before emicizumab initiation to 0.8 (95% CI, 0.47-1.22) after initiation (Table 4). The model-based ABR for treated spontaneous bleeds also decreased, from 1.3 (95% CI, 0.58-3.05) before emicizumab initiation to 0.2 (95% CI, 0.07-0.32) after initiation. For treated joint bleeds, the model-based ABR was 0.5 (95% CI, 0.12-1.73) before emicizumab initiation and 0.2 (95% CI, 0.09-0.35) after initiation. No participants experienced a treated target joint bleed up to week 145. Calculated mean ABRs and calculated median (IQR) ABRs are also reported in Table 4.

**Table 4:** ABR for treated bleeds before and after emicizumab initiation.

Type of annualized bleed rate	Prior to emicizumab initiation^a^ (*n* = 30)	After emicizumab initiation^b^ (*n* = 30)
Treated bleeds		
Model-based ABR (95% CI)^c^	3.6 (2.04-6.46)	0.8 (0.47-1.22)
Model-based ABR ratio (95% CI)	0.21 (0.10-0.42)	
Calculated mean ABR (95% CI)	3.7 (0.94-9.80)	0.7 (0.01-5.10)
Calculated median ABR (IQR)	0.0 (0.00-6.52)	0.4 (0.00-1.09)
Minimum, maximum	0.00, 23.92	0.00, 3.96
Treated joint bleeds		
Model-based ABR (95% CI)^c^	0.5 (0.12-1.73)	0.2 (0.09-0.35)
Model-based ABR ratio (95% CI)	0.39 (0.10-1.55)	
Calculated mean ABR (95% CI)	0.4 (0.00-4.55)	0.2 (0.00-4.04)
Calculated median ABR (IQR)	0.0 (0.00-0.00)	0.0 (0.00-0.33)
Minimum, maximum	0.00, 8.70	0.00, 1.09
Treated target joint bleeds		
Model-based ABR (95% CI)^c^	NE	NE
Model-based ABR ratio (95% CI)	NE	
Calculated mean ABR (95% CI)	0.0 (NA-3.69)	0.0 (NA-3.69)
Calculated median ABR (IQR)	0.0 (0.00-0.00)	0.0 (0.00-0.00)
Minimum, maximum	0.00, 0.00	0.00, 0.00
Treated spontaneous bleeds		
Model-based ABR (95% CI)^c^	1.3 (0.58-3.05)	0.2 (0.07-0.32)
Model-based ABR ratio (95% CI)	0.11 (0.03-0.38)	
Calculated mean ABR (95% CI)	1.3 (0.07-6.09)	0.1 (0.00-3.99)
Calculated median ABR (IQR)	0.0 (0.00-0.00)	0.00 (0.00-0.36)
Minimum, maximum	0.00, 10.87	0.00, 1.44

ABR, annualized bleed rate; NE, not estimable.

a These results are limited by retrospective data collection; prior to emicizumab initiation was defined as 24 weeks prior to enrollment.

b Week 1 of the HOHOEMI/AOZORA study is the date of the first dose of emicizumab and the data cutoff was the last day of week 145 for each participant.

c Estimated using a negative binomial regression model.

## 4 Discussion

No new safety signals were identified in the 3-year interim analysis of AOZORA, which aligns with the outcome from HOHOEMI [2] and confirms the safety profile of emicizumab. Despite prior FVIII prophylaxis in 90% of participants, FVIII inhibitors developed in only 1 participant. In this study, FVIII inhibitor titers were measured every 6 months, ensuring regular monitoring. While it is rare, there have been reports of FVIII inhibitor development beyond 50 exposure days [32,33], suggesting that regular measurement of FVIII inhibitors should be recommended during emicizumab prophylaxis [3]. In addition, the MRI and HJHS data presented in this study are the first to suggest that emicizumab maintains joint health and improves pathologic joint changes in pediatric PwHA.

### 4.1 MRI: synovial hypertrophy and hemosiderin deposition

Repeated joint bleeding causes synovial inflammation and hyperplasia [5,6]; this inflammation-induced neovascularization further increases the risk of bleeding. The cycle of bleeding and inflammation ultimately leads to cartilage degradation and irreversible bone deformity [5,6]. Soft tissue changes on MRI have been reported in joints without symptomatic bleeding [12], and the presence of synovial hypertrophy and hemosiderin on MRI and self-reported bleeding episodes have been shown to predict subsequent osteochondral changes [34]. It has also been reported that joints with synovial hypertrophy on MRI have a significantly higher chance of bleeding over 5 years and that all MRI changes, excluding effusion, can be predictors for the development of arthropathy on radiographs [35]. Hence, early intervention to prevent joint bleeding, including asymptomatic bleeding, is critical to avoid progression to hemophilic arthropathy [10,11]. Synovial hypertrophy and hemosiderin deposition were reported in 10 joints from 7 participants at week 1 in the AOZORA study, including 2 participants aged <2 years (2 individuals received on-demand treatment before entering the AOZORA study, both <1 year of age). MRI findings have been previously observed most frequently in the ankle joints of pediatric PwHA [11], and a similar trend was observed in this study. For those joints in which synovial hypertrophy and hemosiderin deposition were observed at week 1, these findings had disappeared at week 145, and the scores improved. Among the joints where synovial hypertrophy and hemosiderin deposition had disappeared, no joint bleeds occurred between week 1 and week 145, while the joint that showed improved scores had experienced only a single traumatic bleeding episode. Maintaining FVIII activity at 8% to 12% in PwHA with synovial hypertrophy is reported to reduce or resolve synovial hypertrophy [36]. The high trough levels of emicizumab may have contributed to the improvement of synovial hypertrophy and hemosiderin deposition. In the dose- and frequency-escalated Canadian Hemophilia Primary Prophylaxis Study, 54% (25/46) of pediatric participants (initially aged 1.0-2.5 years with normal joints) developed synovial hypertrophy and hemosiderin deposition after 9.6 years of FVIII prophylaxis [34]. In contrast, the AOZORA study (median enrollment age, 4.2 years) showed these findings in only 8% (2/26) of participants at week 145. This lower occurrence rate suggests emicizumab may offer potential advantages for joint health preservation than FVIII products. One participant developed synovial hypertrophy and hemosiderin deposition in the knee joint at week 145. The joint had no MRI findings at week 1 and there were no reports of bleeding requiring treatment from week 1 through week 145, suggesting the possibility of asymptomatic bleeding into the knee joint. However, in the ankle joint of the same participant, the small synovial hypertrophy and hemosiderin deposition confirmed at week 1 had resolved by week 145. The factors causing the ankle and knee joints to undergo different changes in the same participant are unclear; therefore, individual observation of the participant, including their activity levels and lifestyle habits, is necessary.

### 4.2 MRI: effusion/hemarthrosis

At the participant level, no clear trend toward increasing effusion/hemarthrosis was observed. However, at the joint level, the number of joints with these findings increased from week 1 to week 145, which were observed only in participants who were 2 years of age or older at week 1. It is important to note that the IPSG MRI scale does not distinguish between effusion and bleeding. In 5 participants (participants 5-9 in Figure 3) who showed effusion/hemarthrosis with the same score in all joints (knees and ankles) at week 145, the MRI findings may have been a result of physical characteristics rather than bleeding. These 5 participants were <2 to 6 years of age at the time of study entry. No treated bleeding was reported in the joints of these 5 participants where effusion/hemarthrosis was observed from week 1 through week 145. The findings are, therefore, likely associated with effusion/hemarthrosis due to increased physical activity as children grow. Evaluation of MRI findings of the knees and ankles in people with hemophilia and healthy males suggests that joint effusion is not specific to hemophilia [37]. The presence of joint effusion in healthy individuals has also been reported in other studies [38,39], and the presence of effusion has been confirmed by MRI and ultrasound even in healthy children [40]. In the present study, scoring was performed based on the IPSG MRI scale, without distinguishing between effusion and hemarthrosis. In joints where effusion/hemarthrosis was present at week 1, there were few subsequent treated joint bleeds, and no worsening of synovial hypertrophy or hemosiderin deposition was observed at week 145. Even in joints where effusion/hemarthrosis was observed at week 145, there were few reports of treated bleeds from week 1 through week 145. This suggests that many of these findings were effusion rather than hemarthrosis. Based on these observations, we believe that the clinical significance of effusion/hemarthrosis for joint health prognosis is low. However, how joints with these findings change over a long period, and what potential impact they may have on the risk of joint bleeding, will be reassessed in the final analysis.

### 4.3 MRI: osteochondral changes

Osteochondral changes were observed in 2 participants at week 1. In the first participant, who had hemophilic arthropathy, the total score of osteochondral changes decreased due to the absence of subchondral cysts, while the cartilage damage score increased at week 145. Although osteochondral changes can be considered irreversible [6], subchondral cysts have been reported to decrease in volume or naturally regress with normalization of load [41,42]. Meanwhile, cartilage degradation involves multiple pathological processes [43], and it has been suggested that changes in cartilage and subchondral bone occur in parallel [44]. In this participant with hemophilic arthropathy of the ankle, osteochondral changes observed at week 1 were still present at week 145. Once osteochondral changes occur, it is challenging for them to improve [45]. In contrast, bone erosion observed in the left ankle of the second participant at week 1 was no longer present at week 145. Bone erosion is generally considered an irreversible structural change in hemophilic arthropathy. This singular observation raises the possibility that initial bone erosions might potentially be reversible under certain conditions. However, this interim analysis did not include detailed evaluation of changes through image comparison between week 1 and week 145, limiting the ability to fully characterize this phenomenon. Further observation at week 313 may address the question of whether the disappearance of bone erosion is significant or not. Additionally, in the AOZORA study, all joints with synovial hypertrophy and hemosiderin deposition showed improvement and did not progress to osteochondral changes. In the Joint Outcome Study, osteochondral damage was detected by MRI in 2 participants from the prophylaxis group [11]. At the conclusion of the Joint Outcome Continuation study, MRI-detected osteochondral damage was present in 35% of the early prophylaxis group [7], indicating that early prophylaxis was not sufficient to completely prevent joint damage. In contrast, in the AOZORA study, all joints with synovial hypertrophy and hemosiderin deposition showed improvement and did not progress to osteochondral changes, and no participants developed new osteochondral findings by week 145. Despite the 3-year evaluation period, the AOZORA study showed no new osteochondral changes. Emicizumab prophylaxis may potentially inhibit the progression of arthropathy.

### 4.4 Hemophilia Joint Health Score

HJHS is a measure of joint function, and 18 of the 27 participants evaluated through week 145 achieved a score of 0. In the 9 participants who scored higher than 0, strength and extension loss were the most commonly scored domains, with extension loss being the most frequent, especially in the elbow joint. Only 1 joint had an IPSG MRI score and a HJHS higher than 0 at week 145; this was the left ankle joint that was diagnosed with hemophilic arthropathy and showed osteochondral changes; no other joints had scores on both scales at this timepoint. Additionally, 1 knee joint that experienced a joint bleed within 1 month prior to the HJHS assessment showed scores in joint pain, swelling, and duration domains; however, no correlation between joint bleeds and HJHS scores was observed in other joints. Normal values of the HJHS for pediatric people with hemophilia have not been established, and there is no consensus regarding the minimal detectable change for HJHS [46]. Prior studies have defined clinically meaningful changes as ≥4 for total HJHS or ≥2 for individual joint score [[47], [48], [49]]. Although several participants in this study demonstrated HJHS variations of ≥4 points between week 1 and week 145, interpretation of these findings is limited. Since 90% of participants received prophylactic treatment and the average score for individuals with a variable score is less than 1, most participants had no issues with their joint condition and were considered to have relatively well-maintained joints within this study. HJHS may be useful in identifying specific joint changes in individual cases, but it tends to be more valuable in assessing overall deterioration in those with more joint damage than the majority of participants in this study. Additionally, in this interim analysis, no correlation was observed between MRI and HJHS results, suggesting limitations in evaluations that attempt to link MRI and HJHS findings. Each assessment captures different aspects of joint health and using both in combination enables a more comprehensive assessment of joint status.

### 4.5 Limitations

The AOZORA study is a single-arm study of emicizumab with a limited number of participants (*N* = 30). The study included pediatric PwSHA <12 years of age, with participants representing a range of ages at initiation of emicizumab. The age group under 12 years varies widely in age, from infants to school-aged children, making it challenging to evaluate them as a single group; this is due to differences in physical activity and joint stress at various developmental stages.

A total of 90% of participants were receiving prophylactic therapy before the start of emicizumab, but the duration of treatment differed among individuals. MRI scans were performed at each facility according to standardized imaging protocols and then independently evaluated by 2 reviewers centrally; however, while HJHS was conducted using a standardized evaluation method across all facilities and only trained personnel performed the HJHS evaluations, the same evaluator did not always perform sequential assessments, which increases the risk of inter-rater variability [50]. To detect early joint changes, we used MRI evaluation rather than ultrasound. In this study, MRI evaluation of the elbow joints was not conducted to minimize participant burden. Additionally, scoring was conducted according to the IPSG MRI scale, which does not distinguish between effusion and hemarthrosis. Since MRI scans are performed every 3 years, joint changes within the 3 years between scans could not be captured.

## 5 Conclusions

This 3-year interim analysis of the AOZORA study confirms the safety profile of emicizumab and, for the first time, demonstrates the efficacy of emicizumab prophylaxis in maintaining joint health and reversing synovial hypertrophy and hemosiderin deposition in pediatric PwHA. Early therapeutic intervention with emicizumab is crucial, as it can significantly help preserve joint condition. Even in joints with synovial hypertrophy and hemosiderin deposition, reversibility and potential disappearance are anticipated.
