# Supplementary material for: AOZORA study: 3-year interim analysis of safety and joint health in pediatric people with hemophilia A receiving emicizumab prophylaxis
Source: Res Pract Thromb Haemost. 2025 Oct 21;9(8):103228. doi: 10.1016/j.rpth.2025.103228 (PMC12681530; doi:10.1016/j.rpth.2025.103228)
Supplement: Supplementary Tables [file mmc1.docx]

**Supplementary Material**

**Supplementary Table S1.** Imaging procedure manual

**Imaging site**

The following areas should be imaged:

- Bilateral ankle
- Bilateral knee joint

**Imaging conditions**

Magnetic resonance imaging (MRI) scans should be performed under the following imaging conditions:

| Item | Imaging conditions |
| --- | --- |
| Acquisition Sequence | T2*-weighted images |
| Plane of acquisition | - Coronal - Sagittal |
| Parameters in imaging sequences | Perform imaging under the standard imaging conditions set at the site.  Use the following as a guide to set the conditions that do not result in the opposed phase.  For 1.5 T MRI machines   - TR: 400–600 ms - TE: 13.8 ms (recommended) - Flip angle: 20–30°   For 3.0 T MRI machines   - TR: 400–600 ms - TE: 6.9 or 9.2 ms (preferred) - Flip angle: 20–25° |
| Slice thickness | Not more than 5 mm |
| Slice gap | It should be gapless whenever possible. If difficult, a gap of up to 10% should be used. |
| Field-of-view (FOV) | Optimal FOV should be selected based on subject size.  [Reference value] Infants and toddlers: 150 mm; after school age: up to about 180 mm depending on body size |

TR, repetition time; TE, time to echo.

**Recommended conditions**

If possible, images should be taken in the following order: 1. Ankle, 2. Knee

**Supplementary Table S2**. Most common AEs reported by participants.

|  | **Participants entering from HOHOEMI**  **(*n* = 10)** | **Participants newly entering AOZORA**  **(*n* = 20)** | **Total participants**  **(*N* = 30)** |
| --- | --- | --- | --- |
| **Most common AEs affecting ≥20% of participants** |  |  |  |
| Contusion | 10 (100) | 11 (55.0) | 21 (70.0) |
| Nasopharyngitis | 5 (50.0) | 9 (45.0) | 14 (46.7) |
| Gastroenteritis | 4 (40.0) | 4 (20.0) | 8 (26.7) |
| Excoriation | 5 (50.0) | 3 (15.0) | 8 (26.7) |
| Arthralgia | 5 (50.0) | 3 (15.0) | 8 (26.7) |
| Ligament sprain | 5 (50.0) | 2 (10.0) | 7 (23.3) |
| Upper respiratory tract infection | 1 (10.0) | 5 (25.0) | 6 (20.0) |
| Scratch | 6 (60.0) | 0 | 6 (20.0) |
| **AEs affecting ≥2 participants** |  |  |  |
| Nasopharyngitis | 5 (50.0) | 9 (45.0) | 14 (46.7) |
| Gastroenteritis | 4 (40.0) | 4 (20.0) | 8 (26.7) |
| Upper respiratory tract infection | 1 (10.0) | 5 (25.0) | 6 (20.0) |
| Influenza | 5 (50.0) | 0 | 5 (16.7) |
| Otitis media | 2 (20.0) | 1 (5.0) | 3 (10.0) |
| Coronavirus infection | 0 | 2 (10.0) | 2 (6.7) |
| Hordeolum | 0 | 2 (10.0) | 2 (6.7) |
| Impetigo | 0 | 2 (10.0) | 2 (6.7) |
| Otitis media acute | 0 | 2 (10.0) | 2 (6.7) |
| Rhinitis | 0 | 2 (10.0) | 2 (6.7) |
| Viral rash | 0 | 2 (10.0) | 2 (6.7) |
| Contusion | 10 (100) | 11 (55.0) | 21 (70.0) |
| Excoriation | 5 (50.0) | 3 (15.0) | 8 (26.7) |
| Ligament sprain | 5 (50.0) | 2 (10.0) | 7 (23.3) |
| Scratch | 6 (60.0) | 0 | 6 (20.0) |
| Fall | 5 (50.0) | 0 | 5 (16.7) |
| Wound | 3 (30.0) | 2 (10.0) | 5 (16.7) |
| Bite | 2 (20.0) | 0 | 2 (6.7) |
| Mouth injury | 0 | 2 (10.0) | 2 (6.7) |
| Procedural pain | 20 (20.0) | 0 | 2 (6.7) |
| Eczema | 3 (30.0) | 0 | 3 (10.0) |
| Erythema | 1 (10.0) | 1 (5.0) | 2 (6.7) |
| Urticaria | 2 (20.0) | 0 | 2 (6.7) |
| Arthralgia | 5 (50.0) | 3 (15.0) | 8 (26.7) |
| Pain in extremity | 3 (30.0) | 1 (5.0) | 4 (13.3) |
| Oral contusion | 1 (10.0) | 1 (5.0) | 2 (6.7) |
| Vomiting | 0 | 2 (10.0) | 2 (6.7) |
| Injection-site reaction | 1 (10.0) | 4 (20.0) | 5 (16.7) |
| Pyrexia | 1 (10.0) | 2 (10.0) | 3 (10.0) |
| Rhinorrhea | 0 | 2 (10.0) | 2 (6.7) |
| Seasonal allergy | 0 | 2 (10.0) | 2 (6.7) |

AE, adverse event.
